# Supplementary material for: Organic anion transporter 1 is an HDAC4-regulated mediator of nociceptive hypersensitivity in mice
Source: Nat Commun. 2022 Feb 15;13:875. doi: 10.1038/s41467-022-28357-x (PMC8847565; doi:10.1038/s41467-022-28357-x)
Supplement: Supplementary file 2 — Reporting summary [file 41467_2022_28357_MOESM2_ESM.pdf]

nature research

Corresponding author(s): Daniela Mauceri

Last updated by author(s): Jan 12, 2022

Reporting Summary

Nature Research wishes to improve the reproducibility of the work that we publish. This form provides structure for consistency and transparency in reporting. For further information on Nature Research policies, see our Editorial Policies and the Editorial Policy Checklist.

Statistics

For all statistical analyses, confirm that the following items are present in the figure legend, table legend, main text, or Methods section.

n/a	Confirmed	
☐	☒	The exact sample size (n) for each experimental group/condition, given as a discrete number and unit of measurement
☐	☒	A statement on whether measurements were taken from distinct samples or whether the same sample was measured repeatedly
☐	☒	The statistical test(s) used AND whether they are one- or two-sided<br>*Only common tests should be described solely by name; describe more complex techniques in the Methods section.*
☐	☒	A description of all covariates tested
☐	☒	A description of any assumptions or corrections, such as tests of normality and adjustment for multiple comparisons
☐	☒	A full description of the statistical parameters including central tendency (e.g. means) or other basic estimates (e.g. regression coefficient) AND variation (e.g. standard deviation) or associated estimates of uncertainty (e.g. confidence intervals)
☐	☒	For null hypothesis testing, the test statistic (e.g. F, t, r) with confidence intervals, effect sizes, degrees of freedom and P value noted<br>*Give P values as exact values whenever suitable.*
☒	☐	For Bayesian analysis, information on the choice of priors and Markov chain Monte Carlo settings
☒	☐	For hierarchical and complex designs, identification of the appropriate level for tests and full reporting of outcomes
☒	☐	Estimates of effect sizes (e.g. Cohen's d, Pearson's r), indicating how they were calculated

Our web collection on statistics for biologists contains articles on many of the points above.

Software and code

Policy information about availability of computer code

Data collection	StepOne Software 2.3; NIS-Elements AR 4.60; Leica Confocal Software Application Suite X version 3.5.7.23225; VisiView 5.0, Visitron Systems; ChemiDoc Imaging system; Illumina HiSeq - HiSeq Software Suite v3.4.0 for the HiSeq 4000
Data analysis	Microsoft Excel 14.7.7; GraphPad Prism ; IgorPro 8.04; ImageJ 2.0.0-rc-69/1.52u; FIJI 2.3.0/1.53f; Bowtie 2.2.9, TopHat 2.0.14, Cufflinks 2.0.0, Cuffmerge, Cuffdiff 2.0.0.<br>Data analysis detailed information is provided in the Methods section.

For manuscripts utilizing custom algorithms or software that are central to the research but not yet described in published literature, software must be made available to editors and reviewers. We strongly encourage code deposition in a community repository (e.g. GitHub). See the Nature Research guidelines for submitting code & software for further information.

Data

Policy information about availability of data

All manuscripts must include a data availability statement. This statement should provide the following information, where applicable:

- Accession codes, unique identifiers, or web links for publicly available datasets
- A list of figures that have associated raw data
- A description of any restrictions on data availability

RNAseq data generated in this study have been deposited and are available on GEO: GSE159895 (https://www.ncbi.nlm.nih.gov/geo/query/acc.cgi?acc=GSE159895).
All other data presented in this study are provided in the Source Data file associated with this paper.

Field-specific reporting

Please select the one below that is the best fit for your research. If you are not sure, read the appropriate sections before making your selection.

- [x] Life sciences
- [ ] Behavioural & social sciences
- [ ] Ecological, evolutionary & environmental sciences

For a reference copy of the document with all sections, see nature.com/documents/nr-reporting-summary-flat.pdf

Life sciences study design

All studies must disclose on these points even when the disclosure is negative.

Sample size	Sample size were determined according to the existing literature and own experience.<br>doi: 10.1007/s12035-019-01658-x<br>doi: 10.1177/1744806919827469<br>doi: 10.1074/jbc.RA117.001613<br>doi: 10.1074/jbc.M112.432773<br>doi: 10.1016/j.neuron.2012.10.037<br>doi: 10.1038/s41380-019-0491-4
Data exclusions	All data were included in the analyses. Only animals which died during observation time or in surgery were excluded.<br>(CFA-induced thermal and mechanical sensitivity, gene expression LacZ, HDAC4, HDAC4 3SA; CFA-induced thermal and mechanical sensitivity siOat1)
Replication	Each experiment was replicated in independent cohorts of animals (2-3 cohorts, exact N of animals indicated for each experiment) or independent primary cultures (minimum 3, exact n indicated for each experiment).
Randomization	Cultured neuronal dishes were randomly assigned to experimental groups.<br>Mice were randomly assigned to experimental groups. Every cohort or experiment included control and all relative conditions. The order of behavioral analysis was blinded and randomized.
Blinding	Experiments were carried out by a scientist blinded to the tested conditions.

Reporting for specific materials, systems and methods

We require information from authors about some types of materials, experimental systems and methods used in many studies. Here, indicate whether each material, system or method listed is relevant to your study. If you are not sure if a list item applies to your research, read the appropriate section before selecting a response.

Materials & experimental systems

n/a	Involved in the study	
	x	Antibodies
x		Eukaryotic cell lines
x		Palaeontology and archaeology
	x	Animals and other organisms
x		Human research participants
x		Clinical data
x		Dual use research of concern

Methods

n/a	Involved in the study	
x		ChIP-seq
x		Flow cytometry
x		MRI-based neuroimaging

Antibodies

Antibodies used	Rabbit anti-AcH3 Cell signaling Cat# 9649,<br>RRID: AB_823528<br>Rabbit anti-H3 Sigma Aldrich Cat# 06-755,<br>RRID: AB_2118461<br>Mouse anti-Flag Sigma Aldrich Cat# F3165, clone M2<br>RRID: AB_259529<br>Rabbit anti-HA Santa Cruz Cat# sc-805,<br>RRID: AB_631618<br>Rabbit anti-HDAC1 Thermo Fischer Scientific Cat# PA1-860,<br>RRID: AB_2118091<br>Rabbit anti-HDAC3 Cell signaling Cat# 2632,<br>RRID: AB_331545

Rabbit anti-HDAC4 Cell signaling Cat# 7628,
RRID: AB_10860255
Rabbit anti-HDAC5 Cell signaling Cat# 20458,
RRID: AB_2713973
Rabbit anti-HDAC6 Abcam Cat# ab1440,
RRID: AB_2232905
Rabbit anti-HDAC7 Sigma Aldrich Cat# H2662,
RRID: AB_477055
Rabbit anti-HDAC9 Abcam Cat# ab18970,
RRID: AB_470285
Rabbit anti-HDAC10 Abcam Cat# ab53096,
RRID: AB_880349
Rabbit anti-HDAC11 Abcam Cat# ab18973,
RRID: AB_2118240
Mouse anti-NeuN Merck Millipore Cat# MAB377, clone 117724
RRID: AB_2298767
Rabbit anti-OAT1 Abcam Cat# ab135924,
RRID: N/A
Rabbit anti-c-Fos Santa Cruz Cat# sc-52,
RRID: AB_2106783
Mouse anti-β-Actin Santa Cruz Cat# sc-47778, clone C4
RRID: AB_626632
Mouse anti-Tubulin Merck Millipore Cat# T9026, clone DM1A
RRID: AB_477593
Goat-anti-rabbit IgG (H+L) Alexa Fluor® 488 Thermo Fischer Scientific Cat# A11008,
RRID: AB_143165
Goat-anti-mouse IgG (H+L) Alexa Fluor® 594 Thermo Fischer Scientific Cat# A11005,
RRID: AB_141372
Goat anti-mouse IgG (H+L) Peroxidase AffiniPure Jackson Immuno Research Cat# 115-035-003,
RRID: AB_10015289
Goat anti-rabbit IgG (H+L) Peroxidase AffiniPure Jackson Immuno Research Cat# 111-035-144,
RRID: AB_2307391

Validation

Validations are available on the commercial pages of the purchased antibodies.

Rabbit anti-AcH3 Cell signaling Cat# 9649,
https://www.cellsignal.com/products/primary-antibodies/acetyl-histone-h3-lys9-c5b11-rabbit-mab/9649
Acetyl-Histone H3 (Lys9) (C5B11) Rabbit mAb detects endogenous levels of histone H3 only when acetylated on Lys9. This antibody does not cross-react with other acetylated histones. Monoclonal antibody is produced by immunizing animals with a synthetic peptide corresponding to the amino terminus of histone H3 in which Lys9 is acetylated.

Rabbit anti-H3 Sigma Aldrich Cat# 06-755,
https://www.sigmaaldrich.com/IT/it/product/mm/06755
Evaluated by Western Blotting in HeLa acid extract.
Western Blotting Analysis: 0.5 µg/mL of this antibody detected Histone H3 in 10 µg of HeLa acid extract.

Mouse anti-Flag Sigma Aldrich Cat# F3165,
https://www.sigmaaldrich.com/IT/it/product/sigma/f3165
Anti Flag M2 antibody is used for the detection of Flag fusion proteins.This monoclonal antibody is produced in mouse and recognizes the FLAG sequence at the N-terminus, Met N-terminus, and C-terminus. The antibody is also able to recognize FLAG at an internal site. M2, unlike M1 antibody is not Calcium dependent.

Rabbit anti-HA Santa Cruz Cat# sc-805,
https://datasheets.scbt.com/sc-805.pdf

Rabbit anti-HDAC1 Thermo Fischer Scientific Cat# PA1-860,
https://www.thermofisher.com/antibody/product/HDAC1-Antibody-Polyclonal/PA1-860
Antibody specificity was demonstrated by CRISPR-Cas9 mediated knockout of target protein. A loss of signal was observed for target protein in HDAC1 KO cell line compared to control cell line using Anti-HDAC1 Polyclonal Antibody

Rabbit anti-HDAC3 Cell signaling Cat# 2632,
https://www.cellsignal.com/products/primary-antibodies/histone-deacetylase-3-hdac3-antibody/2632
Histone Deacetylase 3 (HDAC3) Antibody detects endogenous levels of total HDAC3 protein. The antibody does not cross-react with other HDAC proteins. Polyclonal antibodies are produced by immunizing animals with a synthetic peptide corresponding to the carboxy-terminal sequence of human HDAC3. Antibodies are purified by protein A and peptide affinity chromatography.

Rabbit anti-HDAC4 Cell signaling Cat# 7628,
v https://www.cellsignal.com/products/primary-antibodies/hdac4-d15c3-rabbit-mab/7628?

_=1640598986383&Ntt=7628&tahead=true
Monoclonal antibody is produced by immunizing animals with a recombinant protein specific to the amino terminus of human HDAC4 protein.

Rabbit anti-HDAC5 Cell signaling Cat# 20458,
https://www.cellsignal.com/products/primary-antibodies/hdac5-d1j7v-rabbit-mab/20458
HDAC5 (D1J7V) Rabbit mAb recognizes endogenous levels of total HDAC5 protein. This antibody does not cross-react with other HDAC proteins, including HDAC4 and HDAC7. Monoclonal antibody is produced by immunizing animals with recombinant protein surrounding Leu445 of human HDAC5 protein.
Rabbit anti-HDAC6 Abcam Cat# ab1440,
https://www.abcam.com/hdac6-antibody-ab1440.html
The antibody detects ~134 kDa histone deacetylase 6. It does not cross-react with other HDAC proteins including HDAC1, 2, 3, 4, 5, 7, and 8.

Rabbit anti-HDAC7 Sigma Aldrich Cat# H2662,
https://www.sigmaaldrich.com/IT/it/product/sigma/h2662?images=true&context=product
Synthetic peptide corresponding to amino acids of human HDAC7, conjugated to KLH.

Rabbit anti-HDAC9 Abcam Cat# ab18970,
https://www.labome.com/product/Abcam/ab18970.html

Rabbit anti-HDAC10 Abcam Cat# ab53096,
https://www.abcam.com/hdac10-antibody-ab53096.html
Synthetic peptide within Human HDAC10 aa 8-53. The exact sequence is proprietary. ab53096detects endogenous levels of total HDAC10 protein.

Rabbit anti-HDAC11 Abcam Cat# ab18973,
https://www.abcam.com/hdac11hd11-antibody-ab18973.html
Synthetic peptide corresponding to Human HDAC11/HD11. ab18973 recognises 39 kDa HDAC-11 of human and mouse origin. It also reacts with rat weakly

Mouse anti-NeuN Merck Millipore Cat# MAB377,
https://www.merckmillipore.com/IT/it/product/Anti-NeuN-Antibody-clone-A60,MM_NF-MAB377?ReferrerURL=https%3A%2F%2Fwww.google.com%2F
Anti-NeuN Antibody, detects level of NeuN and has been published and validated for use in FC, IC, IF, IH, IH(P), IP and WB.

Rabbit anti-OAT1 Abcam Cat# ab135924,
https://www.abcam.com/slc22a6-antibody-ab135924.html
Synthetic peptide derived from internal sequence of Human SLC22A6
Abpromise guarantee covers the use of ab135924 in the following tested applications: ICC, IF, WB

Rabbit anti-c-Fos Santa Cruz Cat# sc-52,
https://www.scbt.com/p/c-fos-antibody-4
epitope mapping at the N-terminus of c-Fos of human origin

Mouse anti-β-Actin Santa Cruz Cat# sc-47778,
https://www.scbt.com/p/beta-actin-antibody-c4?requestFrom=search
Anti-β-Actin Antibody (C4) is a mouse monoclonal IgG1 κ β-Actin antibody, cited in 11,294 publications, provided at 200 µg/ml raised against gizzard Actin of chicken origin
Anti-beta-Actin Antibody (C4) is recommended for detection of β-Actin of mouse, rat, human, avian, bovine, canine, porcine, rabbit, Dictyostelium discoideum and Physarum polycephalum origin by WB, IP, IF, IHC(P) and ELISA; may cross-react with all six known isoforms of Actin in higher vertebrates (including cytoplasmic β- and γ- Actin isoforms, skeletal, cardiac, and vascular α-Actin isoforms, and enteric γ-Actin isoform)

Mouse anti-Tubulin Merck Millipore Cat# T9026,
https://www.sigmaaldrich.com/IT/it/product/sigma/t9026?gclid=Cj0KCQiA5aWOBhDMARIsAIXLlkewfZuEfWZ7JwZxwnhot96j1pcHwT2aMEXUX-Tlm_WVk02F-oykNr0aAqZEEALw_wcB
The antibody is specific for α-tubulin in immunoblotting assays and may be used for localization of α-tubulin in cultured cells or tissue sections. The antibody reacts best with chicken fibroblasts.

Animals and other organisms

Policy information about studies involving animals; ARRIVE guidelines recommended for reporting animal research

Laboratory animals	Adult male or female C57BL/6N wild-type mice between 8 and 14 weeks of age (Charles River) were used throughout all in vivo experiments. For primary neuronal cultures, P0 C57BL/6N mice were used.
Wild animals	The study did not involve wild animals

Field-collected samples	The study did not involve the use of field-collected samples.
Ethics oversight	All animal procedures in this study were carried out in accordance with the ARRIVE guidelines and following approval by the local animal welfare committee (Regierungspräsidium, Karlsruhe, Germany; G3/19). Adult male or female C57BL/6N wild-type mice between 8 and 14 weeks of age (Charles River) were used throughout all in vivo experiments. For primary neuronal cultures, P0 C57BL/6N mice were used. Mice were housed in groups of maximally three animals in standard cages (15 cm x 21 cm x 13.5 cm) within approved animal facilities at Heidelberg University on a 12:12 hours light:dark cycle and maintained at 45-65 % humidity and 20-24 °C with ad libitum access to food and water. Housing conditions were constantly monitored.

Note that full information on the approval of the study protocol must also be provided in the manuscript.
