# Supplementary material for: Organic anion transporter 1 is an HDAC4-regulated mediator of nociceptive hypersensitivity in mice
Source: Nat Commun. 2022 Feb 15;13:875. doi: 10.1038/s41467-022-28357-x (PMC8847565; doi:10.1038/s41467-022-28357-x)
Supplement: Supplementary file 1 — Supplementary Information [file 41467_2022_28357_MOESM1_ESM.pdf]

Supplementary Material

Organic Anion Transporter 1 is an HDAC4-regulated mediator of nociceptive hypersensitivity in mice

Christian Litke, Anna M Hagenston, Ann-Kristin Kenkel, Eszter Paldy, Jianning Lu, Rohini Kuner and Daniela Mauceri

This document contains Supplementary Figures 1-6; Supplementary Table 1, and relative legends.

Supplementary Figure 1

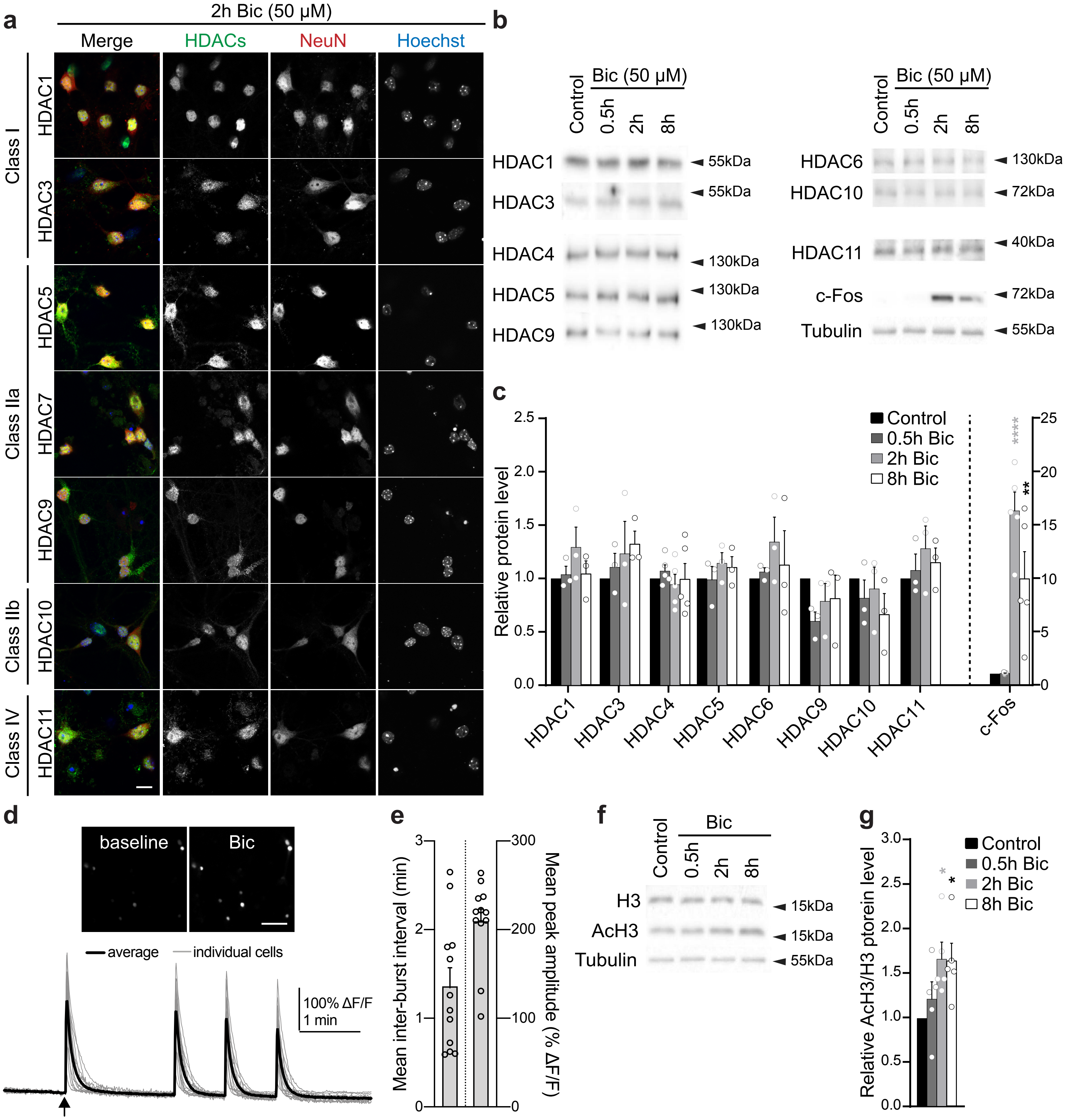

Supplementary Figure 1: Synaptic activity promotes acetylation of histone 3 without affecting total HDACs levels in cultured spinal cord neurons.

(a) Representative images of cultured spinal cord neurons 2 h after Bic application and immunostained for endogenous HDAC1, -3, -5, -7, -9, -10 and -11 (green) and NeuN (red) as neuronal marker. Nuclei were labeled with Hoechst (blue). Scale bar is 20 µm. (b, c) Representative immunoblots and quantification of relative protein levels of the indicated HDACs, c-Fos and tubulin from cultured spinal cord neurons treated with Bic (50 µM) for the indicated durations. Each point represents the mean value derived from one independent experiment (HDAC1 n=3 $F_{(3, 8)}$=1.246; HDAC3 n=3 $F_{(3, 8)}$=0.6382; HDAC4 n=5 $F_{(3, 16)}$=0.3196; HDAC5 n=3 $F_{(3, 8)}$=0.6380; HDAC6 n=3 $F_{(3, 8)}$=0.5612; HDAC9 n=3 $F_{(3, 8)}$=1.270; HDAC10 n=3 $F_{(3, 8)}$=0.7401; HDAC11 n=3 $F_{(3, 8)}$=0.6569; c-Fos n=5 $F_{(3, 16)}$=22.94). (d) Representative images of jRGECO1a-NLS fluorescence prior to and during a calcium transient evoked by Bic (50 µM) and representative traces showing the time course of evoked nuclear calcium transients. (e) Quantification of the inter-peak interval during the first five minutes following Bic application and the amplitude of the first nuclear calcium transient (n=12 coverslips from 3 independent preparations). Scale bar is 50 µm. (f, g) Representative immunoblots and quantification of histone H3, acetylated histone H3-Lys9 (AcH3) and tubulin from spinal cord cultures treated with Bic (50 µM) for the indicated durations (n=5 $F_{(3, 16)}$=3.755). The graph shows the relative AcH3/H3 signal intensity-ratio, normalized to tubulin and controls. In bar graphs, each point represents a value derived from one independent culture. Graphs represent mean ± SEM.

Statistically significant differences were determined by one-way ANOVA followed by Dunnett's post hoc test **, $p < 0.01$; *, $p < 0.05$.

Supplementary Figure 2

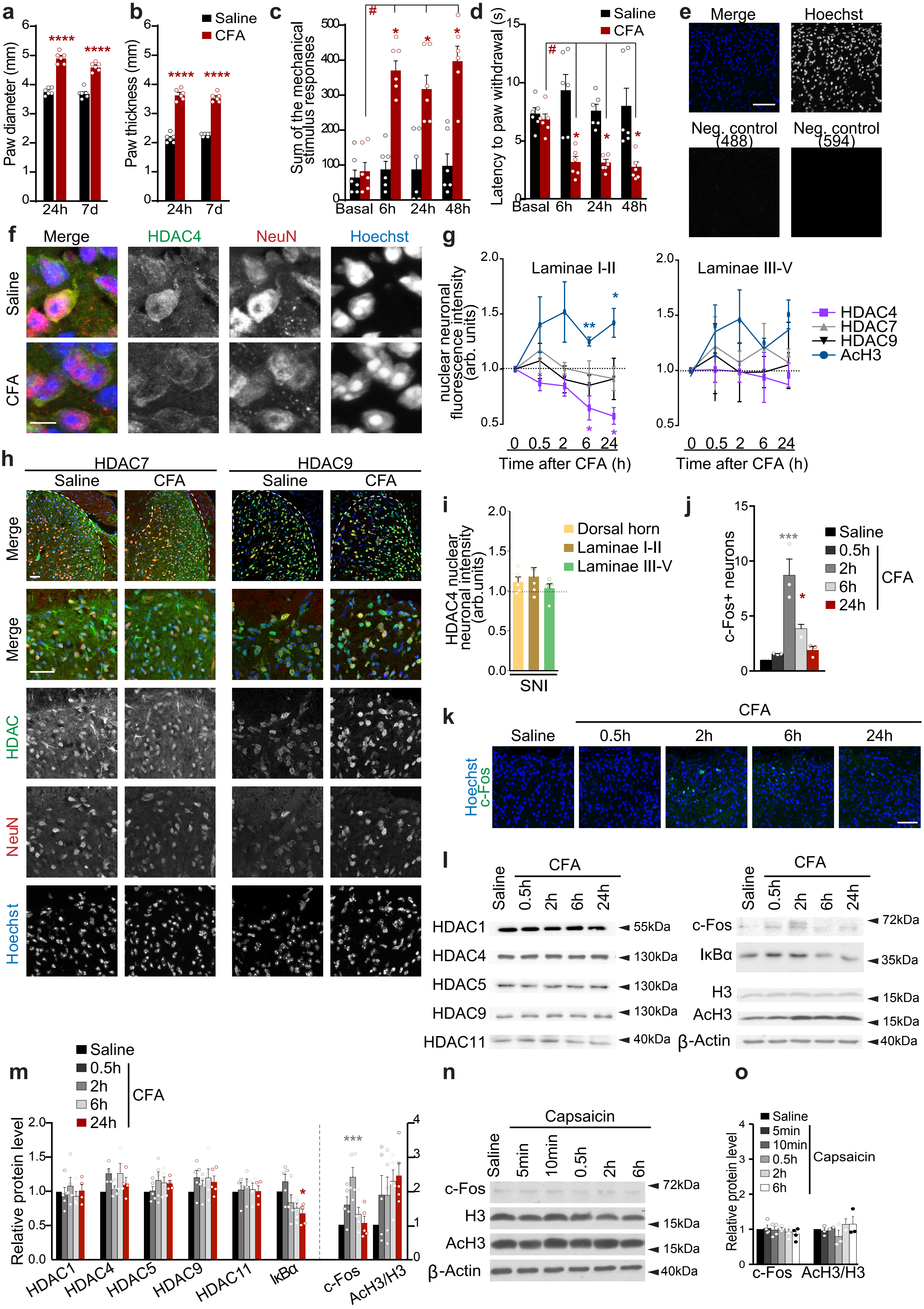

Supplementary Figure 2: Persistent, but not acute, inflammatory nociceptive activity induces the nuclear export of HDAC4 and acetylation of histone 3 in superficial laminae of the spinal cord.

(a) Medial-lateral (diameter) and (b) dorsal-ventral (thickness) dimensions of hind paws from mice after CFA or saline injections (n=6). (c) Mechanical sensitivity after CFA or saline injections, quantified using the sum of stimulus-evoked responses of the paw to von Frey filaments (n=6). (d) Paw withdrawal latency to infrared heat following intraplantar injections as in (c) (n=6). (e) Representative negative control for the experiments shown in Fig. 1e. Scale bar is 60 µm. (f) Representative images of dorsal horn spinal cord neurons immunostained for endogenous HDAC4 (green), neuronal marker NeuN (red); nuclei marker Hoechst (blue). Scale bar is 10 µm. (g) Average nuclear fluorescence intensities from immunolabeled HDACs and AcH3 in dorsal horn neurons, normalized to values from saline-injected mice in superficial or deep laminae (HDAC4 n=4; HDAC7,-9, AcH3 n=5). (h) Representative images of dorsal horn sections of spinal segments L3-5 of mice 24 h after CFA or saline injection, immunostained for HDAC7 or HDAC9 (green), NeuN (red), Hoechst (blue). Higher magnifications of the upper laminae are shown in the lower panels with a scale bar of 40 µm. (i) Relative fluorescence intensity of the nuclear HDAC4 signal in neurons of the lumbar dorsal horn (segments L3-L5), in the superficial (I-II) or deeper laminae (III-V), 21 days after SNI, normalized to sham (n=5). (j) c-Fos-positive neurons in the dorsal horn following injections of saline or CFA (n=3). (k) Representative images of dorsal horn sections of lumbar spinal segments L3-5 of mice injected as in (j), immunostained for c-Fos (green), Hoechst (blue). Scale bar is 60µm. (l, m)

Representative immunoblots and quantification of HDACs, c-Fos, Ikbα, histone H3, AcH3, and b-Actin from dorsal lumbar spinal cord segments L3-5 following saline or CFA injection (HDAC1 n=5 $F_{(4, 19)}$=0.4236; HDAC4 n=4 $F_{(4, 15)}$=2.129; HDAC5 n=5 $F_{(4, 19)}$=1.006; HDAC9 n=5 $F_{(4, 29)}$=1.224; HDAC11 n=4 $F_{(4, 15)}$=0.1333; c-Fos n=5 $F_{(4, 20)}$= 7.446; Ikbα n=5 $F_{(4, 20)}$=1.246; AcH3/H3 n=5 $F_{(4, 20)}$=1.843). (n) Representative immunoblots of c-Fos, H3, AcH3, and b-Actin from dorsal horn tissue of the lumbar spinal cord segments L3-5 following saline or capsaicin injection. (o) Quantification of the relative protein levels as in (n), normalized to values from saline-injected mice. Acetylation of histone H3 is presented as the signal intensity-ratio of AcH3 over H3 (c-Fos n=4 $F_{(5, 18)}$=0.8532; AcH3/H3 n=3 $F_{(5, 10)}$=0.8373). Graphs represent mean ± SEM. In bar graphs, each point represents a value derived from one mouse. Statistically significant differences were determined by one-way ANOVA followed by Dunnett's post hoc test (j, m, o), two-tailed Student's *t*-test (a, b, g, i) or two-way ANOVA with repeated measures followed by Dunnett's post hoc test for comparisons to basal values and multiple *t*-tests for comparisons between saline and CFA (c, d). ****, $p < 0.0001$; ***, $p < 0.001$ **, $p < 0.01$; *, $p < 0.05$; $^{\#}$, $p < 0.001$. Asterisks (*) refer to comparisons between saline and CFA, and hashtags ($^{\#}$) to comparisons relative to basal values in each condition.

Supplementary Figure 3

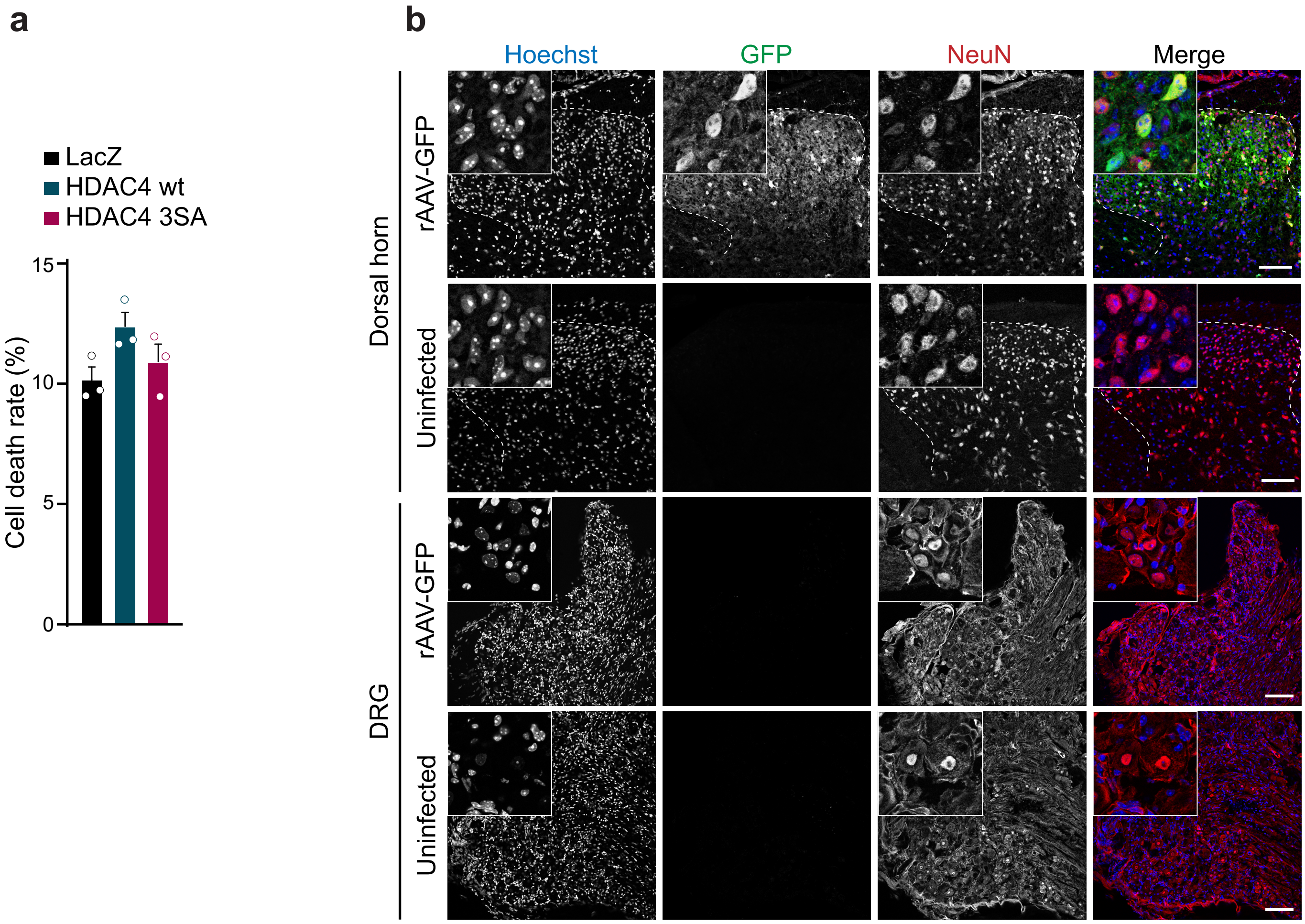

Supplementary Figure 3: Characterization of rAAVs.

(a) Average cell death rate of cultured spinal cord neurons transfected with either LacZ, HDAC4 wt, or HDAC4 3SA. 430 neurons (LacZ), 250 neurons (HDAC4 wt), and 209 neurons (HDAC4 3SA) from three independent experiments were analyzed. (b) Representative images of dorsal horn sections of lumbar spinal cord L3-5 and corresponding dorsal root ganglia (DRG) from mice intra-spinally injected with rAAV-GFP (green) or uninfected and immunostained for NeuN (red) as a neuronal marker. Nuclei were labeled with Hoechst (blue). GFP signal is clearly visible in the dorsal horn but not detectable in the DRG. Scale bars are 100 µm. In bar graphs, each point represents the mean value derived from one independent experiment. Graphs represent mean ± SEM. Statistically significant differences were determined by one-way ANOVA followed by Tukey's post hoc test $F_{(2, 6)}$=3.250.

Supplementary Figure 4

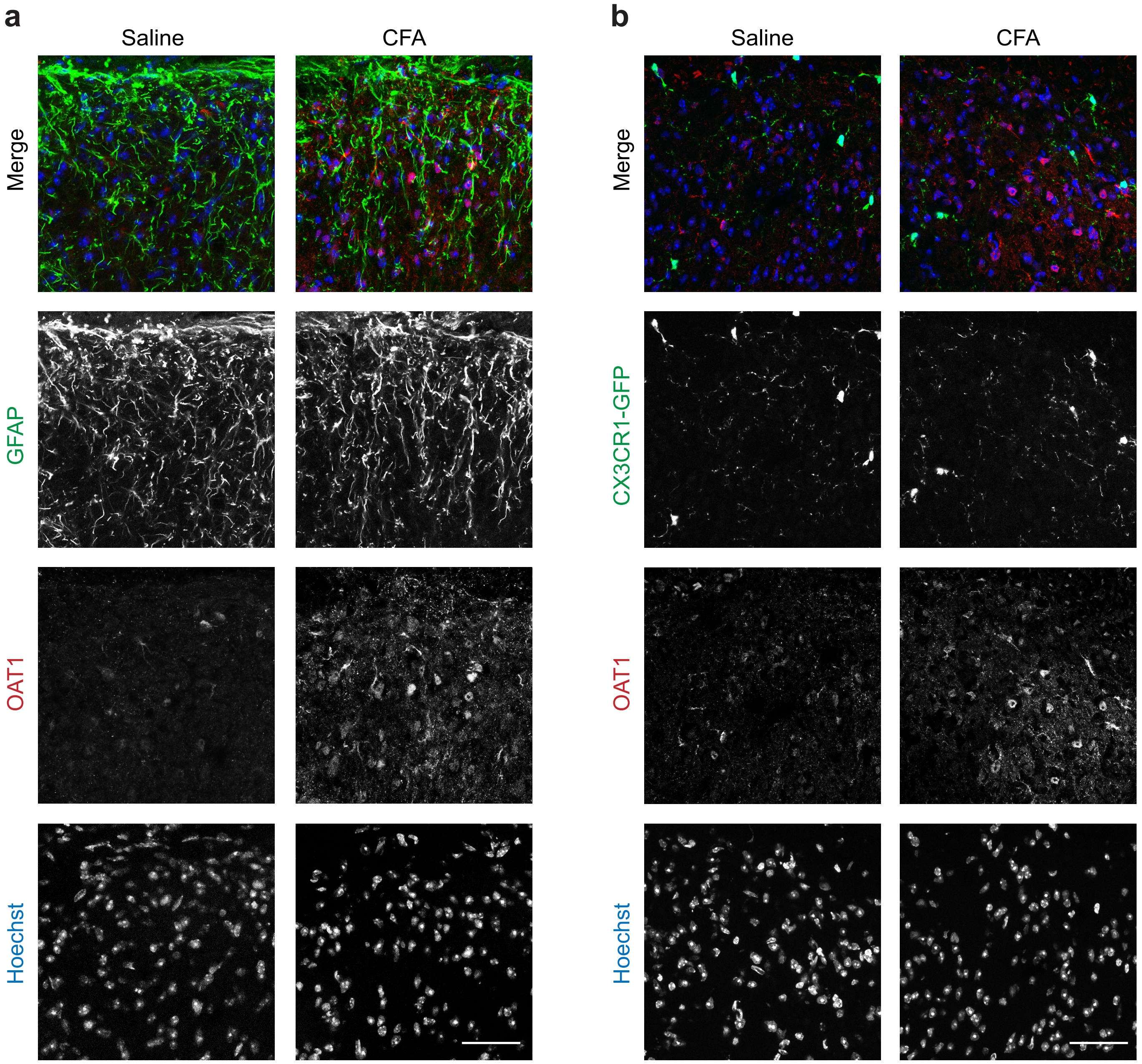

Supplementary Figure 4: OAT1 is not readily detectable in microglia and astrocytes.

(a) Representative images of dorsal horn sections of lumbar spinal cord segments L3-5 of mice 24 h after intraplantar CFA or saline injection, immunostained for the astrocyte marker GFAP (green) and OAT1 (red). Nuclei were labeled with Hoechst (blue). Scale bar is 40 µm. (b) Representative images of dorsal horn sections of lumbar spinal cord segments L3-5 of CXC3R1-GFP mice 24 h after intraplantar CFA or saline injection, GFP (green) is expressed in microglia cells and immunostained for OAT1 (red). Nuclei were labeled with Hoechst (blue). Scale bar is 40 µm.

Supplementary Figure 5

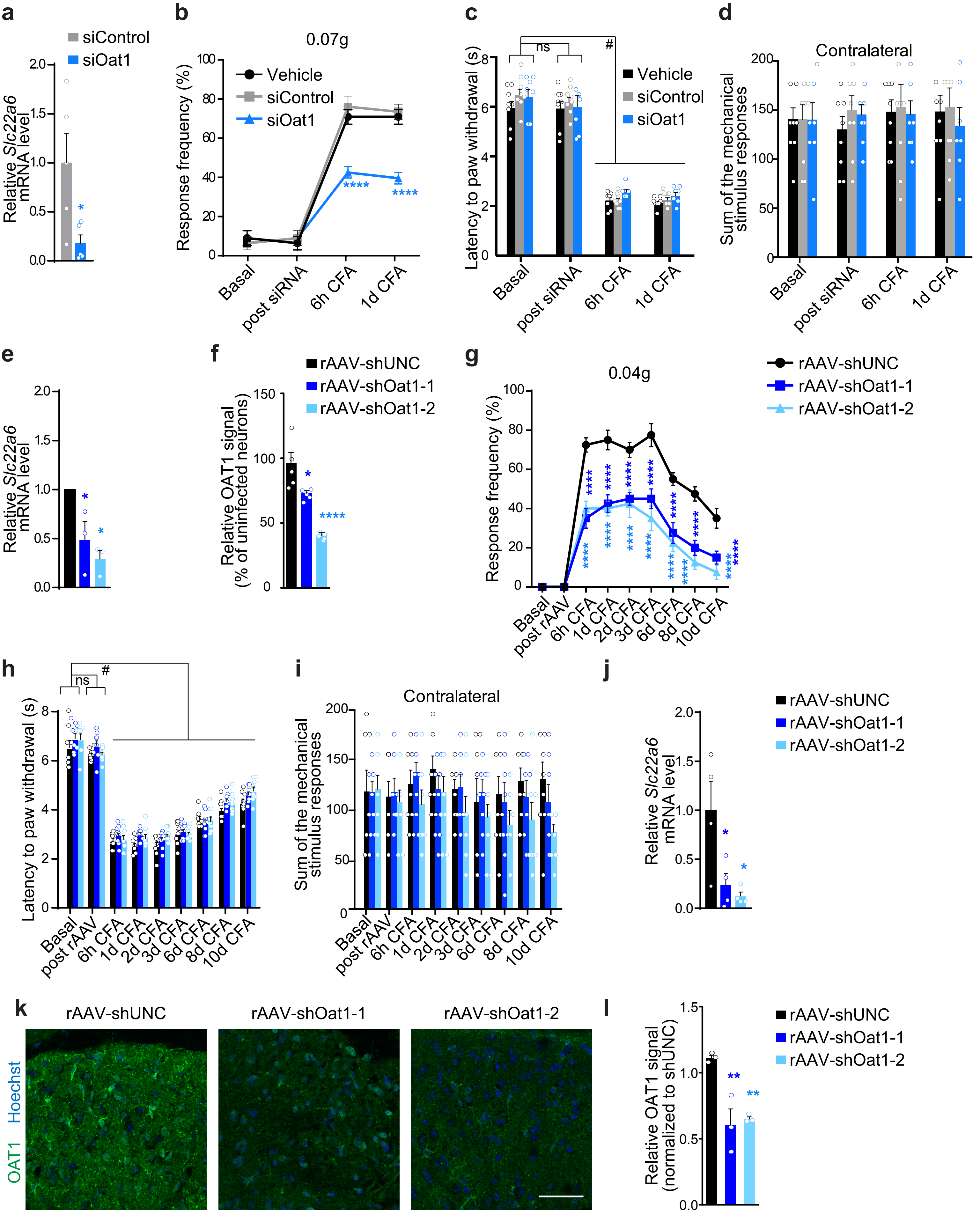

Supplementary Figure 5: *In vivo* acute or prolonged down regulation of OAT1.

(a) QRT-PCR of *Slc22a6* in dorsal lumbar spinal cord segments L3-5, 24 h after the last intrathecal injection of either siControl or siOat1 (n=5). (b) Response frequency to light touch (0.07 g filament) over time. (c) Withdrawal latency of the ipsilateral (CFA-injected) paw to a radiant heat light source. (d) Sum of stimulus-evoked responses to von Frey filaments of the saline-injected (contralateral) paw (b-d Vehicle, siControl n=8 siOat1 n=7). (e) QRT-PCR analysis of *Slc22a6* mRNA in cultured neurons infected with either rAAV-shUNC, rAAV-shOat1-1 or rAAV-shOat1-2 (n=3). (f) Relative OAT1 protein level in cultured neurons (normalized to uninfected cultures, n=5) infected with the indicated rAAVs. (g) Response frequency to light touch (0.04 g filament) over time (n=8 mice). (h) Latency to paw withdrawal to a radiant heat light source for mice intra-spinally injected with rAAVs (n=8). (i) Sum of the responses to mechanical stimulation of the saline-injected (contralateral) paw for mice intra-spinally injected with the indicated rAAVs (n=8). (j) QRT-PCR analysis of *Slc22a6* mRNA *in vivo* in dorsal tissue of the lumbar spinal cord segments L3-5 (n=4). (k) Representative images of dorsal horn sections of lumbar spinal segments L3-5 of mice intra-spinally injected as in (e-j), immunostained for OAT1 (green), Hoechst (blue). Scale bar is 60 µm. (l) Quantification of the relative OAT1 immunofluorescence intensity in the dorsal horn, following intraspinal injections, as in (k) (n=3 mice). In bar graphs, each point represents the mean value derived from one independent mouse. Graphs represent mean ± SEM. Statistically significant differences were determined by two-tailed Student's *t*-test (a), two-way ANOVA with repeated

measures followed by Dunnett's post hoc test (b, $F_{(2, 20)}$=23.18; c, $F_{(2, 20)}$=1.142; d, $F_{(2, 20)}$=0.1083; g, $F_{(2, 21)}$=68.97; h, $F_{(2, 21)}$=3.902; i $F_{(2, 21)}$=1.201), one-way ANOVA followed by Dunnett's post hoc test (e, $F_{(2, 6)}$=9.202347; f, $F_{(2, 12)}$=30.51; j, $F_{(2, 9)}$=6.555337; l $F_{(2, 6)}$=14.76078) ****, $p < 0.0001$; ***, $p < 0.001$; **, $p < 0.01$; *, $p < 0.05$; $^{\#}$, $p < 0.0001$. Asterisks (*) refer to statistical comparisons between conditions and hashtags ($^{\#}$) to comparisons relative to basal values in each condition.

Supplementary Figure 6

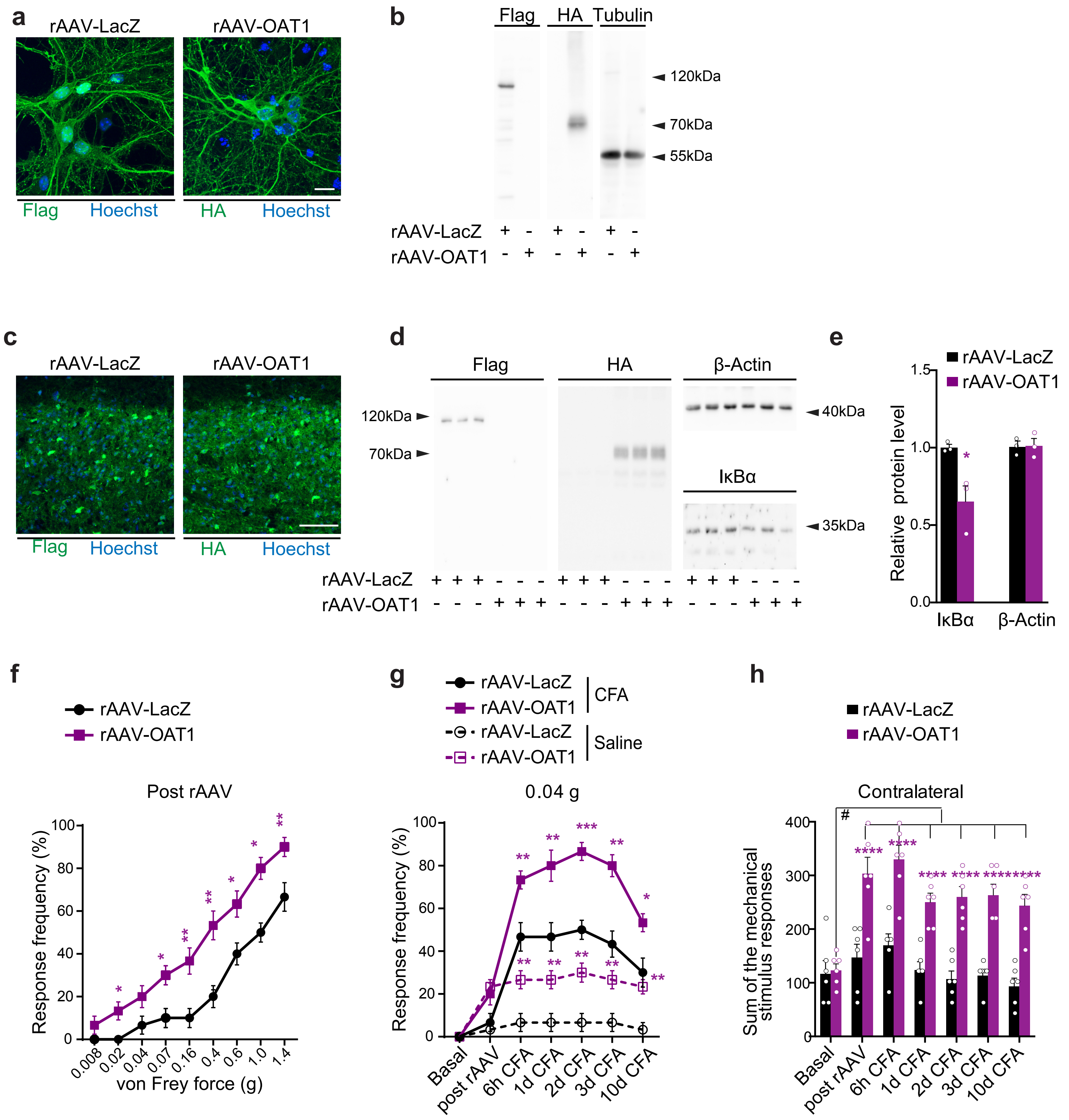

Supplementary Figure 6: In vivo overexpression of OAT1.

(a) Representative images of cultured primary neurons infected with rAAV-LacZ or rAAV-OAT1 and immunostained against their Flag- (rAAV-LacZ) or HA tag (rAAV-OAT1), respectively (green). Hoechst (blue) marks the nuclei. Scale bar is 20 µm. (b) Representative immunoblot of Flag tag, HA tag and tubulin from cultured neurons infected as in (a). (c) Representative images of the dorsal horn from lumbar spinal cord sections L3-5 following intraspinal injections of rAAV-LacZ or rAAV-OAT1 and immunostained against Flag tag (rAAV-LacZ) or HA tag (rAAV-OAT1), respectively (green). Hoechst (blue) marks the nuclei. Scale bar is 60 µm. (d) Representative immunoblots of Flag tag, HA tag, Ikbα and b-Actin from dorsal tissue of the lumbar spinal cord segments L3-5 following intraspinal injections as in (c). (e) Quantification of the relative protein levels of Ik-bα and b-Actin from dorsal tissue of the lumbar spinal cord segments L3-5 following intraspinal injections as in (c, d), normalized to rAAV-LacZ injected control mice (n=3 mice per condition). (f, g) Stimulus intensity-response frequency curves for plantar von Frey mechanical stimulation. Responses to all tested filaments are shown for the basal state after intraspinal rAAV injection (f). Panel (g) shows the response frequency to light touch (0.04 g filament) over time. (h) Sum of stimulus-evoked responses to von Frey filaments of the saline-injected (contralateral) paw for mice intrathecally injected as indicated (n=6 mice per condition (f-h). In bar graphs, each point represents the mean value derived from one independent mouse. Graphs represent mean ± SEM. Statistically significant differences were determined by two-tailed Student's *t*-test (e), or two-way ANOVA with repeated measures followed by Dunnett's post hoc test for comparisons to

basal values and multiple *t*-test with Holm-Sidak correction for comparisons between conditions (f,g,h) ****, $p < 0.0001$; ***, $p < 0.001$; **, $p < 0.01$; *, $p < 0.05$; $^{\#}$, $p < 0.0001$. Asterisks (*) refer to statistical comparisons between conditions and hashtags ($^{\#}$) to comparisons relative to basal values in each condition.

Supplementary Table 1

1. Up-regulated DEGs in inflammatory pain by FC (LacZ-Sal/CFA)

RefSeq ID	Gene symbol	Full gene name	Log2 fold change	FDR adjusted p-value
NM_026862	Cd177	CD177 antigen	4.54	1.51E-02
NM_018857	Msln	mesothelin	4.29	1.51E-02
NM_008766	Slc22a6	solute carrier family 22 (organic anion transporter), member 6	4.13	1.51E-02
NM_010369	Gypa	glycophorin A	3.71	1.51E-02
NM_008694	Ngp	neutrophilic granule protein	3.56	1.51E-02
NM_001281852	S100a9	S100 calcium binding protein A9 (calgranulin B)	3.41	1.51E-02
NM_013650	S100a8	S100 calcium binding protein A8 (calgranulin A)	3.38	1.51E-02
NM_017370	Hp	haptoglobin	3.28	1.51E-02
NM_028770	Krt80	keratin 80	3.26	1.51E-02
NM_009921	Camp	cathelicidin antimicrobial peptide	3.15	1.51E-02
NM_008491	Lcn2	lipocalin 2	3.07	1.51E-02
NM_009264	Sprr1a	small proline-rich protein 1A	3.03	1.51E-02
NM_145947	Slc26a7	solute carrier family 26, member 7	2.98	1.51E-02
NM_008522	Ltf	lactotransferrin	2.95	1.51E-02
NM_008685	Nfe2	nuclear factor, erythroid derived 2	2.95	3.79E-02
NM_001033281	Prdm6	PR domain containing 6	2.78	1.51E-02
NM_011403	Slc4a1	solute carrier family 4 (anion exchanger), member 1	2.75	1.51E-02
NM_011410	Slfn4	schlafen 4	2.71	1.51E-02
NM_009892	Chil3	chitinase-like 3	2.65	1.51E-02
NM_008611	Mmp8	matrix metallopeptidase 8	2.62	1.51E-02
NM_008599	Cxcl9	chemokine (C-X-C motif) ligand 9	2.61	1.51E-02
NM_181596	Retnlg	resistin like gamma	2.43	1.51E-02
NM_001099217	Ly6c2	lymphocyte antigen 6 complex, locus C2	2.29	4.63E-02
NM_029796	Lrg1	leucine-rich alpha-2-glycoprotein 1	2.17	1.51E-02
NM_026743	Tspan11	tetraspanin 11	2.08	1.51E-02
NM_001080971	Tubb1	tubulin, beta 1 class VI	2.07	2.64E-02
NM_007759	Crabp2	cellular retinoic acid binding protein II	1.92	1.51E-02
NM_023785	Ppbp	pro-platelet basic protein	1.89	1.51E-02
NM_007655	Cd79a	CD79A antigen (immunoglobulin-associated alpha)	1.82	1.51E-02
NM_007876	Dpep1	dipeptidase 1	1.74	1.51E-02
NM_010855	Myh4	myosin, heavy polypeptide 4, skeletal muscle	1.67	1.51E-02
NR_130974	H19	H19, imprinted maternally expressed transcript	1.53	1.51E-02
NM_010730	Anxa1	annexin A1	1.42	1.51E-02
NM_025806	Plbd1	phospholipase B domain containing 1	1.39	1.51E-02
NM_007962	Mpzl2	myelin protein zero-like 2	1.39	1.51E-02
NM_008339	Cd79b	CD79B antigen	1.35	4.63E-02
NM_010545	Cd74	CD74 antigen	1.29	1.51E-02
NM_001081117	Mki67	antigen identified by monoclonal antibody Ki 67	1.27	1.51E-02
NM_010930	Nov	nephroblastoma overexpressed gene	1.25	1.51E-02
NM_013519	Foxc2	forkhead box C2	1.23	1.51E-02
NM_010382	H2-Eb1	histocompatibility 2, class II antigen E beta	1.20	1.51E-02
NM_011859	Osr1	odd-skipped related transcription factor 1	1.19	1.51E-02

NM_009606	Acta1	actin, alpha 1, skeletal muscle	1.18	1.51E-02
NM_010378	H2-Aa	histocompatibility 2, class II antigen A, alpha	1.18	1.51E-02
NM_207105	H2-Ab1	histocompatibility 2, class II antigen A, beta 1	1.18	1.51E-02
NM_001253874	Itgal	integrin alpha L	1.17	2.64E-02
NM_008489	Lbp	lipopolysaccharide binding protein	1.16	1.51E-02
NM_008009	Fgfbp1	fibroblast growth factor binding protein 1	1.15	1.51E-02
NM_008147	Lilr4b	leukocyte immunoglobulin-like receptor, subfamily B, member 4B	1.13	4.63E-02
NM_172479	Slc38a5	solute carrier family 38, member 5	1.09	1.51E-02
NM_001313914	Thbs1	thrombospondin 1	1.08	1.51E-02
NM_198024	Ranbp3l	RAN binding protein 3-like	1.07	1.51E-02
NM_001033374	Gm694	steroid receptor associated and regulated protein	1.05	4.63E-02
NM_008963	Ptgds	prostaglandin D2 synthase (brain)	1.04	1.51E-02
NM_198095	Bst2	bone marrow stromal cell antigen 2	1.01	1.51E-02
NM_001177954	Rpgr	retinitis pigmentosa GTPase regulator	1.00	2.64E-02
NM_011255	Rbp4	retinol binding protein 4, plasma	0.99	1.51E-02
NM_001286502	Pdyn	prodynorphin	0.98	1.51E-02
NM_013706	Cd52	CD52 antigen	0.96	3.79E-02
NM_001145799	Ctla2a	cytotoxic T lymphocyte-associated protein 2 alpha	0.96	1.51E-02
NM_001276413	Fn1	fibronectin 1	0.96	1.51E-02
NM_172892	Slc13a4	solute carrier family 13 (sodium/sulfate symporters), member 4	0.94	1.51E-02
NM_008597	Mgp	matrix Gla protein	0.93	1.51E-02
NM_010582	Itih2	inter-alpha trypsin inhibitor, heavy chain 2	0.92	2.64E-02
NM_009526	Wnt6	wingless-type MMTV integration site family, member 6	0.91	1.51E-02
NM_008344	Igfbp6	insulin-like growth factor binding protein 6	0.91	1.51E-02
NM_009008	Rac2	Rac family small GTPase 2	0.91	1.51E-02
NM_001143689	H2-Q4	histocompatibility 2, Q region locus 4	0.89	1.51E-02
NM_010728	Lox	lysyl oxidase	0.88	1.51E-02
NM_001201391	Hbb-bs	hemoglobin, beta adult s chain	0.88	1.51E-02
NM_139142	Slc6a20a	solute carrier family 6 (neurotransmitter transporter), member 20A	0.87	1.51E-02
NM_010260	Gbp2	guanylate binding protein 2	0.86	1.51E-02
NM_153170	Slc36a2	solute carrier family 36 (proton/amino acid symporter), member 2	0.85	1.51E-02
NM_008036	Fosb	FBJ osteosarcoma oncogene B	0.85	4.63E-02
NM_009378	Thbd	thrombomodulin	0.83	1.51E-02
NM_016907	Spint1	serine protease inhibitor, Kunitz type 1	0.82	3.79E-02
NM_007486	Arhgdib	Rho, GDP dissociation inhibitor (GDI) beta	0.82	2.64E-02
NM_001168318	Scara5	scavenger receptor class A, member 5	0.81	1.51E-02
NM_011355	Spi1	spleen focus forming virus (SFFV) proviral integration oncogene	0.81	4.63E-02
NM_011708	Vwf	Von Willebrand factor	0.77	1.51E-02
NM_009864	Cdh1	cadherin 1	0.76	1.51E-02
NM_010118	Egr2	early growth response 2	0.74	1.51E-02
NM_019412	Prx	periaxin	0.72	1.51E-02
NM_148927	Plekha4	pleckstrin homology domain containing, family A, member 4	0.71	1.51E-02
NM_172604	Scara3	scavenger receptor class A, member 3	0.70	2.64E-02
NM_001013741	Ddn	dendrin	0.70	1.51E-02
NM_007857	Dhh	desert hedgehog	0.68	1.51E-02
NM_027957	Fam178b	family with sequence similarity 178, member B	0.67	1.51E-02
NM_001001892	H2-K1	histocompatibility 2, K1, K region	0.67	1.51E-02

NM_015776	Mfap5	microfibrillar associated protein 5	0.67	1.51E-02
NM_001291539	Sema3b	semaphorin 3B	0.66	1.51E-02
NM_012043	Islr	immunoglobulin superfamily containing leucine-rich repeat	0.64	2.64E-02
NM_009369	Tgfbi	transforming growth factor, beta induced	0.63	1.51E-02
NM_008623	Mpz	myelin protein zero	0.63	1.51E-02
NM_010078	Drp2	dystrophin related protein 2	0.63	1.51E-02
NM_007557	Bmp7	bone morphogenetic protein 7	0.63	1.51E-02
NM_007742	Col1a1	collagen, type I, alpha 1	0.63	1.51E-02
NM_008885	Pmp22	peripheral myelin protein 22	0.59	1.51E-02
NM_153105	Cldn19	claudin 19	0.59	2.64E-02
NM_010380	H2-D1	histocompatibility 2, D region locus 1	0.59	1.51E-02
NM_080553	Itpr3	inositol 1,4,5-triphosphate receptor 3	0.54	1.51E-02
NM_008760	Ogn	osteoglycin	0.53	2.64E-02
NM_027907	Etnppl	ethanolamine phosphate phospholyase	0.51	4.63E-02
NM_008305	Hspg2	perlecan (heparan sulfate proteoglycan 2)	0.50	4.63E-02

2. Down-regulated DEGs in inflammatory pain by FC (LacZ-Sal/CFA)

RefSeq ID	Gene symbol	Full gene name	Log2 fold change	FDR adjusted p-value
NM_028735	Ttc21a	tetratricopeptide repeat domain 21A	-0.95	2.64E-02
NM_001081369	Ccdc153	coiled-coil domain containing 153	-1.21	1.51E-02
NM_030187	Ak7	adenylate kinase 7	-1.21	1.51E-02
NM_001163638	Cfap70	cilia and flagella associated protein 70	-1.33	4.63E-02
NM_177922	Mapk15	mitogen-activated protein kinase 15	-1.44	1.51E-02
NM_177629	Fam216b	family with sequence similarity 216, member B	-1.50	1.51E-02
NM_001164669	Dnah6	dynein, axonemal, heavy chain 6	-1.54	1.51E-02
NM_198660	Ccdc180	coiled-coil domain containing 180	-1.59	4.63E-02
NM_029122	Iqca	IQ motif containing with AAA domain	-1.70	1.51E-02

3. Up-regulated DEGs in inflammatory pain by FC (HDAC4 wt-Sal/CFA)

RefSeq ID	Gene symbol	Full gene name	Log2 fold change	FDR adjusted p-value
NM_026183	Slc47a1	solute carrier family 47, member 1	4.99	1.26E-02
NM_001168680	Tspan8	tetraspanin 8	3.87	1.26E-02
NM_016685	Comp	cartilage oligomeric matrix protein	3.79	1.26E-02
NM_145947	Slc26a7	solute carrier family 26, member 7	3.21	1.26E-02
NM_018857	Msln	mesothelin	3.01	1.26E-02
NM_008766	Slc22a6	solute carrier family 22 (organic anion transporter), member 6	2.68	1.26E-02
NR_130974	H19	H19, imprinted maternally expressed transcript	2.50	1.26E-02
NM_029796	Lrg1	leucine-rich alpha-2-glycoprotein 1	2.02	1.26E-02
NM_170727	Scgb3a1	secretoglobin, family 3A, member 1	1.99	3.47E-02
NM_009854	Cd7	CD7 antigen	1.78	1.26E-02
NM_026743	Tspan11	tetraspanin 11	1.77	1.26E-02
NM_008491	Lcn2	lipocalin 2	1.66	1.26E-02
NM_011910	Uts2	urotensin 2	1.44	1.26E-02

NM_021712	Slc18a3	solute carrier family 18 (vesicular monoamine), member 3	1.36	1.26E-02
NM_001313914	Thbs1	thrombospondin 1	1.36	1.26E-02
NM_013639	Prph	peripherin	1.31	1.26E-02
NM_029568	Mfap4	microfibrillar-associated protein 4	1.26	1.26E-02
NM_173403	Slc10a4	solute carrier family 10 (sodium/bile acid cotransporter family), member 4	1.25	1.26E-02
NM_009504	Vdr	vitamin D (1,25-dihydroxyvitamin D3) receptor	1.24	2.45E-02
NM_009264	Sprr1a	small proline-rich protein 1A	1.12	4.53E-02
NM_139134	Chodl	chondrolectin	1.11	1.26E-02
NM_145963	Kcnj14	potassium inwardly-rectifying channel, subfamily J, member 14	1.09	1.26E-02
NM_008489	Lbp	lipopolysaccharide binding protein	1.08	1.26E-02
NM_022025	Slc5a7	solute carrier family 5 (choline transporter), member 7	1.05	1.26E-02
NM_008597	Mgp	matrix Gla protein	1.00	1.26E-02
NM_010846	Mx1	MX dynamin-like GTPase 1	0.95	1.26E-02
NM_013653	Ccl5	chemokine (C-C motif) ligand 5	0.84	1.26E-02
NM_010930	Nov	nephroblastoma overexpressed gene	0.81	3.47E-02
NM_021487	Kcne1l	potassium voltage-gated channel, Isk-related family, member 1	0.81	1.26E-02
NM_013560	Hspb1	heat shock protein 1	0.80	1.26E-02
NM_001276413	Fn1	fibronectin 1	0.78	1.26E-02
NM_001289444	Calca	calcitonin/calcitonin-related polypeptide, alpha	0.76	4.53E-02
NM_001161845	Sgk1	serum/glucocorticoid regulated kinase 1	0.74	1.26E-02
NM_021384	Rsad2	radical S-adenosyl methionine domain containing 2	0.73	2.45E-02
NM_011426	Siglec1	sialic acid binding Ig-like lectin 1, sialoadhesin	0.73	1.26E-02
NM_146007	Col6a2	collagen, type VI, alpha 2	0.73	3.47E-02
NM_025734	Kcng4	potassium voltage-gated channel, subfamily G, member 4	0.69	1.26E-02
NM_011708	Vwf	Von Willebrand factor	0.69	1.26E-02
NM_001252601	Irf7	interferon regulatory factor 7	0.67	1.26E-02
NM_010904	Nefh	neurofilament, heavy polypeptide	0.67	1.26E-02
NM_053110	Gpnmb	glycoprotein (transmembrane) nmb	0.64	1.26E-02
NM_009778	C3	complement component 3	0.62	1.26E-02
NM_013723	Podxl	podocalyxin-like	0.59	1.26E-02
NM_009252	Serpina3n	serine (or cysteine) peptidase inhibitor, clade A, member 3N	0.56	1.26E-02
NM_008630	Mt2	metallothionein 2	0.55	2.45E-02
NM_011854	Oasl2	2'-5' oligoadenylate synthetase-like 2	0.52	4.53E-02
NM_013602	Mt1	metallothionein 1	0.52	1.26E-02

4. Down-regulated DEGs in inflammatory pain by FC (HDAC4 wt-Sal/CFA)

RefSeq ID	Gene symbol	Full gene name	Log2 fold change	FDR adjusted p-value
NM_008694	Ngp	neutrophilic granule protein	-1.24	3.47E-02
NM_009606	Acta1	actin, alpha 1, skeletal muscle	-1.95	1.26E-02
NM_001013767	Capn11	calpain 11	-2.07	1.26E-02
NM_001163669	Tnnt3	troponin T3, skeletal, fast	-2.56	1.26E-02
NM_013697	Ttr	transthyretin	-4.67	1.26E-02

5. Up-regulated DEGs in inflammatory pain by FC (HDAC4 3SA-Sal/CFA)

RefSeq ID	Gene symbol	Full gene name	Log2 fold change	FDR adjusted p-value
NM_009690	Cd5l	CD5 antigen-like	2.50	1.47E-02
NM_009608	Actc1	actin, alpha, cardiac muscle 1	2.46	2.79E-02
NM_053188	Srd5a2	steroid 5 alpha-reductase 2	2.20	8.50E-03
NM_011315	Saa3	serum amyloid A 3	2.09	2.79E-02
NM_134160	Mcoln3	mucolipin 3	2.04	8.50E-03
NM_001199940	Serpina3i	serine (or cysteine) peptidase inhibitor, clade A, member 3I	1.98	2.79E-02
NM_011910	Uts2	urotensin 2	1.96	8.50E-03
NM_175406	Atp6v0d2	ATPase, H+ transporting, lysosomal V0 subunit D2	1.87	8.50E-03
NM_019944	Mnx1	motor neuron and pancreas homeobox 1	1.86	8.50E-03
NM_011426	Siglec1	sialic acid binding Ig-like lectin 1, sialoadhesin	1.85	8.50E-03
NM_145226	Oas3	2'-5' oligoadenylate synthetase 3	1.76	8.50E-03
NM_018729	Cd244	CD244 molecule A	1.72	3.13E-02
NM_207244	Cd200r4	CD200 receptor 4	1.65	8.50E-03
NM_024406	Fabp4	fatty acid binding protein 4, adipocyte	1.65	3.54E-02
NM_008491	Lcn2	lipocalin 2	1.63	8.50E-03
NM_001310331	Ms4a4a	membrane-spanning 4-domains, subfamily A, member 4A	1.62	8.50E-03
NM_010819	Clec4d	C-type lectin domain family 4, member d	1.56	3.54E-02
NM_001164327	Phf11b	PHD finger protein 11B	1.55	8.50E-03
NM_008486	Anpep	alanyl (membrane) aminopeptidase	1.53	8.50E-03
NM_011407	Slfn1	schlafen 1	1.53	8.50E-03
NM_029796	Lrg1	leucine-rich alpha-2-glycoprotein 1	1.52	8.50E-03
NM_152803	Hpse	heparanase	1.50	8.50E-03
NM_001277944	Apoc2	apolipoprotein C-II	1.49	3.13E-02
NM_145209	Oasl1	2'-5' oligoadenylate synthetase-like 1	1.49	8.50E-03
NM_013639	Prph	peripherin	1.48	8.50E-03
NM_008372	Il7r	interleukin 7 receptor	1.48	1.47E-02
NM_009977	Cst7	cystatin F (leukocystatin)	1.47	8.50E-03
NM_001204203	Spp1	secreted phosphoprotein 1	1.44	8.50E-03
NM_001165932	Ucma	upper zone of growth plate and cartilage matrix associated	1.44	1.47E-02
NM_144955	Nkx6-1	NK6 homeobox 1	1.43	8.50E-03
NM_145227	Oas2	2'-5' oligoadenylate synthetase 2	1.43	8.50E-03
NM_009890	Ch25h	cholesterol 25-hydroxylase	1.43	1.47E-02
NM_001177350	Pydc4	interferon activated gene 213	1.42	2.79E-02
NM_001081957	Wfdc17	WAP four-disulfide core domain 17	1.41	4.55E-02
NM_001113326	Msr1	macrophage scavenger receptor 1	1.39	8.50E-03
NM_010705	Lgals3	lectin, galactose binding, soluble 3	1.38	8.50E-03
NM_027397	Isl2	insulin related protein 2 (islet 2)	1.38	3.91E-02
NM_001204910	AI607873	interferon activated gene 207	1.37	8.50E-03
NM_001004174	AA467197	expressed sequence AA467197	1.37	1.96E-02
NM_017372	Lyz2	lysozyme 2	1.36	8.50E-03
NM_021384	Rsad2	radical S-adenosyl methionine domain containing 2	1.36	8.50E-03
NM_001024230	Gm5431	predicted gene 5431	1.34	4.55E-02
NM_011819	Gdf15	growth differentiation factor 15	1.34	1.96E-02
NR_110993	Cd68	CD68 antigen	1.32	8.50E-03

NM_009891	Chat	choline acetyltransferase	1.31	3.91E-02
NM_010846	Mx1	MX dynamin-like GTPase 1	1.31	8.50E-03
NM_008147	Lilr4b	leukocyte immunoglobulin-like receptor, subfamily B, member 4B	1.31	8.50E-03
NM_029499	Ms4a4c	membrane-spanning 4-domains, subfamily A, member 4C	1.31	8.50E-03
NM_001276398	Ms4a7	membrane-spanning 4-domains, subfamily A, member 7	1.30	1.47E-02
NM_009264	Sprr1a	small proline-rich protein 1A	1.30	4.94E-02
NM_175026	Pyhin1	interferon activated gene 209	1.29	8.50E-03
NM_018866	Cxcl13	chemokine (C-X-C motif) ligand 13	1.29	8.50E-03
NM_021394	Zbp1	Z-DNA binding protein 1	1.29	1.96E-02
NM_145211	Oas1a	2'-5' oligoadenylate synthetase 1A	1.28	8.50E-03
NM_021718	Ms4a4b	membrane-spanning 4-domains, subfamily A, member 4B	1.27	1.96E-02
NM_021712	Slc18a3	solute carrier family 18 (vesicular monoamine), member 3	1.26	1.96E-02
NM_001252601	Irf7	interferon regulatory factor 7	1.23	8.50E-03
NM_001163256	Fblim1	filamin binding LIM protein 1	1.23	8.50E-03
NM_026835	Ms4a6d	membrane-spanning 4-domains, subfamily A, member 6D	1.22	8.50E-03
NM_007807	Cybb	cytochrome b-245, beta polypeptide	1.21	8.50E-03
NM_030707	Fcrls	Fc receptor-like S, scavenger receptor	1.21	8.50E-03
NM_144830	Tmem106a	transmembrane protein 106A	1.20	8.50E-03
NM_009403	Tnfsf8	tumor necrosis factor (ligand) superfamily, member 8	1.19	3.13E-02
NM_008873	Plau	plasminogen activator, urokinase	1.19	8.50E-03
NM_010821	Mpeg1	macrophage expressed gene 1	1.19	8.50E-03
NM_026829	Mthfs	5, 10-methenyltetrahydrofolate synthetase	1.18	2.44E-02
NM_009635	Avil	advillin	1.18	4.26E-02
NM_177337	Arl11	ADP-ribosylation factor-like 11	1.18	2.44E-02
NM_001167743	Slfn8	schlafen 8	1.17	8.50E-03
NM_001276413	Fn1	fibronectin 1	1.17	8.50E-03
NM_009845	Cd22	CD22 antigen	1.17	1.96E-02
NM_008329	Ifi204	interferon activated gene 204	1.16	1.47E-02
NM_153098	Cd109	CD109 antigen	1.16	8.50E-03
NM_001291066	Adam8	a disintegrin and metallopeptidase domain 8	1.16	2.79E-02
NM_001286610	Arhgap25	Rho GTPase activating protein 25	1.15	8.50E-03
NM_021334	Itgax	integrin alpha X	1.15	8.50E-03
NM_170758	Cd300a	CD300A molecule	1.15	2.44E-02
NM_001038654	Slc16a3	solute carrier family 16, member 3	1.15	2.44E-02
NM_011662	Tyrobp	TYRO protein tyrosine kinase binding protein	1.15	8.50E-03
NM_011198	Ptgs2	prostaglandin-endoperoxide synthase 2	1.14	4.26E-02
NM_010871	Naip6	NLR family, apoptosis inhibitory protein 6	1.14	3.91E-02
NR_003508	Mx2	MX dynamin-like GTPase 2	1.14	3.13E-02
NM_011408	Slfn2	schlafen 2	1.13	8.50E-03
NM_007781	Csf2rb2	colony stimulating factor 2 receptor, beta 2, low-affinity	1.13	1.96E-02
NM_172796	Slfn9	schlafen 9	1.13	1.47E-02
NM_001302650	Ifi203	interferon activated gene 203	1.12	1.96E-02
NM_008489	Lbp	lipopolysaccharide binding protein	1.12	3.13E-02
NM_031254	Trem2	triggering receptor expressed on myeloid cells 2	1.11	8.50E-03
NM_206536	AB124611	cDNA sequence AB124611	1.11	4.55E-02
NM_010186	Fcgr1	Fc receptor, IgG, high affinity I	1.10	8.50E-03
NM_008404	Itgb2	integrin beta 2	1.10	8.50E-03

NM_031159	Apobec1	apolipoprotein B mRNA editing enzyme, catalytic polypeptide 1	1.10	1.47E-02
NM_008533	Cd180	CD180 antigen	1.10	8.50E-03
NM_013560	Hspb1	heat shock protein 1	1.10	8.50E-03
NM_001290758	Tlr7	toll-like receptor 7	1.10	8.50E-03
NM_008369	Il3ra	interleukin 3 receptor, alpha chain	1.10	4.26E-02
NM_011909	Usp18	ubiquitin specific peptidase 18	1.10	8.50E-03
NM_013653	Ccl5	chemokine (C-C motif) ligand 5	1.10	3.13E-02
NM_031376	Pik3ap1	phosphoinositide-3-kinase adaptor protein 1	1.10	8.50E-03
NM_013489	Cd84	CD84 antigen	1.09	8.50E-03
NM_010501	Ifit3	interferon-induced protein with tetratricopeptide repeats 3	1.08	8.50E-03
NM_009099	Trim30a	tripartite motif-containing 30A	1.08	8.50E-03
NM_001277968	Ly9	lymphocyte antigen 9	1.08	8.50E-03
NM_023380	Samsn1	SAM domain, SH3 domain and nuclear localization signals, 1	1.08	3.13E-02
NM_173403	Slc10a4	solute carrier family 10 (sodium/bile acid cotransporter family), member 4	1.08	3.13E-02
NM_019738	Nupr1	nuclear protein transcription regulator 1	1.08	4.55E-02
NM_008153	Cmklr1	chemokine-like receptor 1	1.08	2.44E-02
NM_019984	Tgm1	transglutaminase 1, K polypeptide	1.08	4.26E-02
NM_008353	Il12rb1	interleukin 12 receptor, beta 1	1.07	4.26E-02
NM_011723	Xdh	xanthine dehydrogenase	1.07	8.50E-03
NM_007498	Atf3	activating transcription factor 3	1.06	8.50E-03
NM_013706	Cd52	CD52 antigen	1.06	8.50E-03
NM_020008	Clec7a	C-type lectin domain family 7, member a	1.06	8.50E-03
NM_001081123	Arhgap36	Rho GTPase activating protein 36	1.06	8.50E-03
NM_008332	Ifit2	interferon-induced protein with tetratricopeptide repeats 2	1.06	8.50E-03
NM_001190161	Psrc1	proline/serine-rich coiled-coil 1	1.05	8.50E-03
NM_030150	Dhx58	DEXH (Asp-Glu-X-His) box polypeptide 58	1.05	1.96E-02
NM_009779	C3ar1	complement component 3a receptor 1	1.05	8.50E-03
NM_011539	Tbxas1	thromboxane A synthase 1, platelet	1.05	2.79E-02
NM_205820	Tlr13	toll-like receptor 13	1.05	8.50E-03
NM_007574	C1qc	complement component 1, q subcomponent, C chain	1.05	8.50E-03
NM_022964	Lat2	linker for activation of T cells family, member 2	1.04	1.96E-02
NM_001308535	Il2rg	interleukin 2 receptor, gamma chain	1.04	1.47E-02
NM_145509	Rab7b	RAB7B, member RAS oncogene family	1.04	8.50E-03
NM_009777	C1qb	complement component 1, q subcomponent, beta polypeptide	1.04	8.50E-03
NM_011176	St14	suppression of tumorigenicity 14 (colon carcinoma)	1.04	8.50E-03
NM_183201	Slfn5	schlafen 5	1.03	8.50E-03
NM_009139	Ccl6	chemokine (C-C motif) ligand 6	1.03	2.79E-02
NM_013482	Btk	Bruton agammaglobulinemia tyrosine kinase	1.03	3.54E-02
NM_019388	Cd86	CD86 antigen	1.03	2.44E-02
NM_130449	Colec12	collectin sub-family member 12	1.02	8.50E-03
NM_008528	Blnk	B cell linker	1.02	3.13E-02
NM_007645	Cd37	CD37 antigen	1.02	2.44E-02
NM_001146022	Wdfy4	WD repeat and FYVE domain containing 4	1.02	1.47E-02
NM_001033767	Gm4951	predicted gene 4951	1.02	1.47E-02
NM_007806	Cyba	cytochrome b-245, alpha polypeptide	1.02	8.50E-03
NM_133871	Ifi44	interferon-induced protein 44	1.01	8.50E-03
NM_001146275	Iigp1	interferon inducible GTPase 1	1.01	8.50E-03

NM_013612	Slc11a1	solute carrier family 11 (proton-coupled divalent metal ion transporters), member 1	1.01	1.47E-02
NM_008175	Grn	granulin	1.01	8.50E-03
NM_011815	Fyb	FYN binding protein	1.01	8.50E-03
NM_011150	Lgals3bp	lectin, galactoside-binding, soluble, 3 binding protein	1.01	8.50E-03
NM_007780	Csf2rb	colony stimulating factor 2 receptor, beta, low-affinity	1.01	1.96E-02
NM_001039530	Parp14	poly (ADP-ribose) polymerase family, member 14	1.00	8.50E-03
NM_015783	Isg15	ISG15 ubiquitin-like modifier	1.00	3.13E-02
NM_010686	Laptm5	lysosomal-associated protein transmembrane 5	1.00	8.50E-03
NM_001005858	Ifit3b	interferon-induced protein with tetratricopeptide repeats 3B	1.00	1.47E-02
NM_009911	Cxcr4	chemokine (C-X-C motif) receptor 4	1.00	2.79E-02
NM_053214	Myo1f	myosin IF	0.99	8.50E-03
NM_013820	Hk2	hexokinase 2	0.99	1.96E-02
NM_134250	Havcr2	hepatitis A virus cellular receptor 2	0.99	2.44E-02
NM_001099634	Myof	myoferlin	0.98	3.91E-02
NM_022325	Ctsz	cathepsin Z	0.98	8.50E-03
NM_001033308	Themis2	thymocyte selection associated family member 2	0.98	4.55E-02
NM_021281	Ctss	cathepsin S	0.98	8.50E-03
NM_178785	Rasal3	RAS protein activator like 3	0.98	1.47E-02
NM_007572	C1qa	complement component 1, q subcomponent, alpha polypeptide	0.98	8.50E-03
NM_013792	Naglu	alpha-N-acetylglucosaminidase (Sanfilippo disease IIIB)	0.97	8.50E-03
NM_153564	Gbp5	guanylate binding protein 5	0.96	1.47E-02
NM_198095	Bst2	bone marrow stromal cell antigen 2	0.96	8.50E-03
NM_021451	Pmaip1	phorbol-12-myristate-13-acetate-induced protein 1	0.95	2.44E-02
NM_134158	Cd300c2	CD300C molecule 2	0.95	1.96E-02
NM_001145960	Slc37a2	solute carrier family 37, member 2	0.95	1.96E-02
NM_011355	Spi1	spleen focus forming virus (SFFV) proviral integration oncogene	0.95	1.47E-02
NM_174857	Mamdc2	MAM domain containing 2	0.95	4.55E-02
NM_001162500	Parvg	parvin, gamma	0.95	8.50E-03
NM_008320	Irf8	interferon regulatory factor 8	0.95	8.50E-03
NM_001033207	Nlrc5	NLR family, CARD domain containing 5	0.94	1.96E-02
NM_153505	Nckap1l	NCK associated protein 1 like	0.94	8.50E-03
NM_001313712	Sp100	nuclear antigen Sp100	0.94	3.54E-02
NM_010368	Gusb	glucuronidase, beta	0.94	1.47E-02
NM_007651	Cd53	CD53 antigen	0.94	8.50E-03
NM_001253874	Itgal	integrin alpha L	0.94	4.26E-02
NM_013470	Anxa3	annexin A3	0.93	8.50E-03
NM_010156	Samd9l	sterile alpha motif domain containing 9-like	0.93	1.96E-02
NM_011019	Osmr	oncostatin M receptor	0.93	1.47E-02
NM_001126182	Naip2	NLR family, apoptosis inhibitory protein 2	0.93	3.13E-02
NM_013545	Ptpn6	protein tyrosine phosphatase, non-receptor type 6	0.92	1.96E-02
NM_183162	Helz2	helicase with zinc finger 2, transcriptional coactivator	0.92	8.50E-03
NM_009252	Serpina3n	serine (or cysteine) peptidase inhibitor, clade A, member 3N	0.91	8.50E-03
NM_001005508	Arhgap30	Rho GTPase activating protein 30	0.91	1.96E-02
NM_010745	Ly86	lymphocyte antigen 86	0.91	8.50E-03
NM_001033342	Cdc42bpg	CDC42 binding protein kinase gamma (DMPK-like)	0.91	8.50E-03
NM_001247984	Lcp1	lymphocyte cytosolic protein 1	0.91	8.50E-03
NM_008161	Gpx3	glutathione peroxidase 3	0.90	8.50E-03

NM_175088	Mdfic	MyoD family inhibitor domain containing	0.90	4.55E-02
NM_009778	C3	complement component 3	0.89	8.50E-03
NM_008225	Hcls1	hematopoietic cell specific Lyn substrate 1	0.89	3.13E-02
NM_001142952	Fam46c	terminal nucleotidyltransferase 5C	0.89	8.50E-03
NM_007801	Ctsh	cathepsin H	0.89	8.50E-03
NM_001190830	Jak3	Janus kinase 3	0.88	4.94E-02
NM_010130	Adgre1	adhesion G protein-coupled receptor E1	0.88	1.47E-02
NM_027521	Hmha1	Rho GTPase activating protein 45	0.88	1.47E-02
NM_008401	Itgam	integrin alpha M	0.88	8.50E-03
NM_010493	Icam1	intercellular adhesion molecule 1	0.87	2.44E-02
NM_001161111	Pqlc3	PQ loop repeat containing	0.87	4.55E-02
NM_010398	H2-T23	histocompatibility 2, T region locus 23	0.87	8.50E-03
NM_028195	Cyth4	cytohesin 4	0.86	8.50E-03
NM_010833	Msn	moesin	0.86	8.50E-03
NM_011854	Oasl2	2'-5' oligoadenylate synthetase-like 2	0.85	8.50E-03
NM_001289848	Cox6b2	cytochrome c oxidase subunit 6B2	0.85	4.55E-02
NM_028785	Dock8	dedicator of cytokinesis 8	0.85	1.47E-02
NM_010658	Mafb	v-maf musculoaponeurotic fibrosarcoma oncogene family, protein B (avian)	0.85	8.50E-03
NM_027835	Ifih1	interferon induced with helicase C domain 1	0.85	2.79E-02
NM_172285	Plcg2	phospholipase C, gamma 2	0.84	4.55E-02
NM_010260	Gbp2	guanylate binding protein 2	0.84	1.47E-02
NM_010240	Ftl1	ferritin light polypeptide 1	0.84	8.50E-03
NM_172689	Ddx58	DEAD (Asp-Glu-Ala-Asp) box polypeptide 58	0.83	2.79E-02
NM_010421	Hexa	hexosaminidase A	0.83	8.50E-03
NM_001163815	Vav1	vav 1 oncogene	0.83	4.55E-02
NM_007408	Plin2	perilipin 2	0.82	1.96E-02
NM_008331	Ifit1	interferon-induced protein with tetratricopeptide repeats 1	0.82	2.79E-02
NM_138672	Stab1	stabilin 1	0.82	2.44E-02
NM_001161730	Tap1	transporter 1, ATP-binding cassette, sub-family B (MDR/TAP)	0.82	3.54E-02
NM_010188	Fcgr3	Fc receptor, IgG, low affinity III	0.82	1.47E-02
NM_010422	Hexb	hexosaminidase B	0.82	8.50E-03
NM_001287514	Cebpa	CCAAT/enhancer binding protein (C/EBP), alpha	0.81	3.54E-02
NM_023141	Tor3a	torsin family 3, member A	0.80	2.79E-02
NM_001143689	H2-Q4	histocompatibility 2, Q region locus 4	0.80	1.47E-02
NM_001001892	H2-K1	histocompatibility 2, K1, K region	0.80	1.47E-02
NM_019449	Unc93b1	unc-93 homolog B1, TLR signaling regulator	0.80	3.54E-02
NM_009546	Trim25	tripartite motif-containing 25	0.79	4.55E-02
NM_008326	Irgm1	immunity-related GTPase family M member 1	0.79	1.96E-02
NM_178911	Pld4	phospholipase D family, member 4	0.78	2.79E-02
NM_001110192	Inpp5d	inositol polyphosphate-5-phosphatase D	0.78	3.13E-02
NM_018738	Igtp, Irgm2	interferon gamma induced GTPase	0.78	2.79E-02
NM_001162366	Ptk2b	PTK2 protein tyrosine kinase 2 beta	0.78	2.79E-02
NM_025378	Ifitm3	interferon induced transmembrane protein 3	0.77	4.26E-02
NM_011610	Tnfrsf1b	tumor necrosis factor receptor superfamily, member 1b	0.77	3.91E-02
NM_010380	H2-D1	histocompatibility 2, D region locus 1	0.77	8.50E-03
NM_001083312	Gbp7	guanylate binding protein 7	0.76	1.96E-02
NM_011609	Tnfrsf1a	tumor necrosis factor receptor superfamily, member 1a	0.76	1.96E-02

NM_011708	Vwf	Von Willebrand factor	0.76	1.96E-02
NM_009982	Ctsc	cathepsin C	0.75	2.79E-02
NM_007669	Cdkn1a	cyclin-dependent kinase inhibitor 1A (P21)	0.75	2.79E-02
NM_001271446	Ly6a	lymphocyte antigen 6 complex, locus A	0.75	4.26E-02
NM_009735	B2m	beta-2 microglobulin	0.75	2.79E-02
NM_010764	Man2b1	mannosidase 2, alpha B1	0.73	1.47E-02
NM_023409	Npc2	NPC intracellular cholesterol transporter 2	0.73	3.91E-02
NM_001164036	Ly6e	lymphocyte antigen 6 complex, locus E	0.73	3.91E-02
NM_026428	Dcxr	dicarbonyl L-xylulose reductase	0.73	3.13E-02
NM_007798	Ctsb	cathepsin B	0.71	4.55E-02
NM_001190974	Axl	AXL receptor tyrosine kinase	0.71	2.79E-02
NM_013454	Abca1	ATP-binding cassette, sub-family A (ABC1), member 1	0.71	2.44E-02
NM_010145	Ephx1	epoxide hydrolase 1, microsomal	0.70	3.91E-02
NM_010580	Itgb5	integrin beta 5	0.69	2.44E-02
NM_008630	Mt2	metallothionein 2	0.68	4.94E-02
NM_011701	Vim	vimentin	0.68	3.54E-02
NM_009987	Cx3cr1	chemokine (C-X3-C motif) receptor 1	0.68	3.13E-02
NM_029364	Gns	glucosamine (N-acetyl)-6-sulfatase	0.67	3.13E-02
NM_013602	Mt1	metallothionein 1	0.66	3.54E-02
NM_009831	Ccng1	cyclin G1	0.66	4.94E-02

6. Down-regulated DEGs in inflammatory pain by FC (HDAC4 3SA-Sal/CFA)

**RefSeq ID**	**Gene symbol**	**Full gene name**	**Log2 fold change**	**FDR adjusted p-value**
NM_008623	Mpz	myelin protein zero	-0.74	2.79E-02
NM_019412	Prx	periaxin	-0.75	8.50E-03
NM_145555	Ncmap	noncompact myelin associated protein	-0.91	3.13E-02
NM_009135	Scn7a	sodium channel, voltage-gated, type VII, alpha	-1.17	8.50E-03
NM_028572	Vgll3	vestigial like family member 3	-1.19	2.79E-02
NM_008694	Ngp	neutrophilic granule protein	-1.30	2.44E-02

Supplementary Table 1:

Lists of up-regulated and down-regulated differentially expressed genes (DEGs) 24 h after intraplantar CFA injection. Tables are organized by expressed transgene (LacZ, HDAC4 wt, HDAC4 3SA) and show RefSeq ID, Gene symbol, Full gene name, Log2 fold change and FDR adjusted *p*-value. FC= fold change. All data are additionally available on GEO: GSE159895.
